# Limited Secondary Transmission of SARS-CoV-2 in Child Care Programs — Rhode Island, June 1–July 31, 2020

**DOI:** 10.15585/mmwr.mm6934e2

**Published:** 2020-08-28

**Authors:** Ruth Link-Gelles, Amanda L. DellaGrotta, Caitlin Molina, Ailis Clyne, Kristine Campagna, Tatiana M. Lanzieri, Marisa A. Hast, Krishna Palipudi, Emilio Dirlikov, Utpala Bandy

**Affiliations:** ^1^CDC COVID-19 Response Team; ^2^Rhode Island Department of Health; ^3^Rhode Island Department of Human Services; ^4^Epidemic Intelligence Service, CDC.

On June 1, 2020, with declines in coronavirus disease 2019 (COVID-19) cases and hospitalizations in Rhode Island,* child care programs in the state reopened after a nearly 3-month closure implemented as part of mitigation efforts. To reopen safely, the Rhode Island Department of Human Services (RIDHS) required licensed center- and home-based child care programs to reduce enrollment, initially to a maximum of 12 persons, including staff members, in stable groups (i.e., staff members and students not switching between groups) in physically separated spaces, increasing to a maximum of 20 persons on June 29. Additional requirements included universal use of masks for adults, daily symptom screening of adults and children, and enhanced cleaning and disinfection according to CDC guidelines.^†^ As of July 31, 666 of 891 (75%) programs were approved to reopen, with capacity for 18,945 children, representing 74% of the state’s January 2020 child care program population (25,749 children).

High compliance with RIDHS requirements was observed during 127 unannounced program monitoring visits (C Molina, RIDHS, personal communication, 2020). Program administrators reported that maintaining stable staffing was the most difficult requirement to implement because of the need to rotate staff members to cover teacher breaks, vacation, and sick leave and that continued adherence to small, stable classes might not be feasible without additional funding.

During June 1–July 31, the Rhode Island Department of Health (RIDOH) conducted investigations of any reported COVID-19 case in a child or adult, including staff members, parents, or guardians, present at a child care program. Reported cases were classified as confirmed if a person received a positive reverse transcription–polymerase chain reaction (RT-PCR) test result for SARS-CoV-2, the virus that causes COVID-19, or probable if a person met clinical and epidemiologic criteria with no laboratory testing.^§^ Child care classes with a symptomatic person identified were required to close for 14 days or until the case could be ruled out by a negative RT-PCR test result. RIDOH quarantined contacts^¶^ and conducted symptom monitoring via a weekly phone call or daily text message; symptomatic contacts were referred for testing.

A total of 101 possible child care–associated COVID-19 cases were reported during June 1–July 31. Among them, 49 (49%) symptomatic persons were excluded after receiving negative laboratory test results, 33 persons (33%) had confirmed cases, and 19 (19%) were classified as having probable cases. Among the 52 confirmed and probable cases, 30 (58%) were among children (median age = 5 years), and 22 (42%) were among adults (20 teachers and two parents [median age = 30 years]) (Table). Overall, 39 (75%) cases occurred from mid- to late July, when incidence in the state was increasing (Figure). Cases were confirmed a median of 2 days (range = 0–11 days) after specimen collection. The identification of 101 possible child care–associated COVID-19 cases resulted in closures of 89 classes and quarantine of 687 children and 166 staff members, including contacts.

**TABLE:** Child care–associated confirmed and probable COVID-19 cases (N = 52)* — Rhode Island, June 1–July 31, 2020

Characteristic	Case classification, no (%)					
	Children, n = 30 (58)			Adults, n = 22 (42)		
	All cases	Confirmed	Probable	All cases	Confirmed	Probable
**No. of cases**	30	17 (57)	13 (43)	22	16 (73)	6 (27)
**Sex**						
Female	16 (53)	11 (64)	5 (38)	21 (95)	15 (94)	6 (100)
Male	14 (47)	6 (36)	8 (62)	1 (5)	1 (6)	0 (0)
**Age, yrs, median (range)** ^†,§,¶^	5 (0.5–12)	5 (0.5–12)	4 (1–5)	30 (20–63)	32 (20–60)	30 (20–63)
**Days from specimen collection to report to RIDOH, median (range)**	2 (0–8)	2 (0–8)	N/A	3 (0–11)	3 (0–11)	N/A

**Abbreviations:** COVID-19 = coronavirus disease 2019; N/A = not applicable; RIDOH = Rhode Island Department of Health.

* Includes all cases considered index and secondary transmission. Reported cases were classified as confirmed if a person received a positive reverse transcription–polymerase chain reaction SARS-CoV-2 test result or probable if a person met clinical and epidemiologic criteria, with no laboratory testing.

^†^ Age was missing for three children with probable COVID-19.

^§^ Age was missing for two adults with probable COVID-19.

^¶^ In Rhode Island, children up to age 12 years are permitted to attend child care programs during the summer; use of a mask is not currently required for any child in child care.

**FIGURE:**
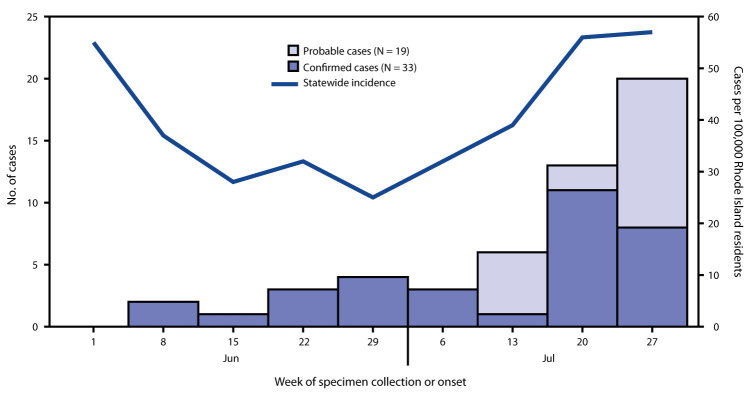
Child care–associated confirmed (N = 33) and probable (N = 19) COVID-19 cases,* by specimen collection or onset week^†^ and incidence of confirmed COVID-19 cases^§^ — Rhode Island, June 1–July 31, 2020 **Abbreviation:** COVID-19 = coronavirus disease 2019. * Confirmed cases were defined as a positive reverse transcription–polymerase chain reaction test result for SARS-CoV-2, the virus that causes COVID-19; probable cases met clinical and epidemiologic criteria, with no laboratory testing. ^†^ Probable cases did not have specimens collected and are therefore listed by symptom onset date. ^§^ Data on incidence were sourced via Rhode Island Department of Health and include confirmed cases only.

Cases occurred in 29 child care programs, 20 (69%) of which had a single case with no apparent secondary transmission. Five (15%) programs had two to five cases; however, RIDOH excluded child care–related transmission because of the timing of symptom onset. In late June, a child aged 2 years attended child care for 6 days while potentially infectious, including 3 days before symptom onset (parent-reported fever to 100.3°F [37.9°C] and chills) and 3 days after symptom resolution. Ten of 11 child care contacts were tested for SARS-CoV-2 a median of 2 days after last exposure (range = 1–3 days); none had a positive test result. Epidemiologic investigation by RIDOH indicated adherence to RIDHS regulations.

Secondary transmission in four child care programs after July 15 could not be ruled out. In one program, RIDOH epidemiologic investigation identified lack of adherence to RIDHS regulations, including switching between groups. Ten confirmed cases (five children, four staff members, and one parent) were identified among contacts in the program. The program was closed, and 60 children and 21 staff members were quarantined for 14 days. In the second program, three confirmed cases were identified from a single classroom; 26 students and 17 staff members were quarantined. The third program had two cases with symptom onset dates indicating potential transmission; however, no epidemiologic link was identified. The fourth program had two cases, one in a staff member and the other in a child contact of the staff member. The staff member moved among all classrooms, exposing adults and children in the entire program, which was subsequently closed; 37 students and 16 staff members were quarantined.

Rhode Island reopened child care programs in the context of low SARS-CoV-2 transmission relative to other U.S. states. Possible secondary transmission was identified in four of the 666 programs that had been allowed to reopen, all in the last 2 weeks of July, when community transmission in Rhode Island increased. The apparent absence of secondary transmission within the other 662 child care programs was likely the result of RIDOH response efforts to contain transmission and child care programs’ adherence to RIDHS requirements, in particular maximum class sizes and use of face masks for adults (*1*). However, case ascertainment among children is challenging, given high rates of asymptomatic infection or mild disease (*2*,*3*), and SARS-CoV-2 infections were likely undetected. Despite limited identified secondary transmission, the impact on child care programs was substantial, with 853 children and staff members quarantined, which highlights the importance of community mitigation efforts to safeguard child care programs. Adherence to current CDC recommendations remains critical to reducing transmission in child care settings, including wearing of masks by adults, limiting mixing between established student-teacher groups, staying home when ill, and cleaning and disinfecting frequently touched surfaces.** Timely public health action, including case investigation and contact tracing, is critical to minimizing outbreaks in child care programs.^††^

## Footnotes

* https://www.bostonglobe.com/2020/05/29/metro/new-covid-19-cases-hospitalizations-fall-ri-governor-gives-update-friday/.

† https://www.cdc.gov/coronavirus/2019-ncov/community/schools-childcare/guidance-for-childcare.html#CleanDisinfect.

§ https://wwwn.cdc.gov/nndss/conditions/coronavirus-disease-2019-covid-19/case-definition/2020/.

¶ https://reopeningri.com/wp-content/uploads/2020/07/Child-Care-Playbook-07232020_vShared_5-002-2.pdf.

** https://www.cdc.gov/coronavirus/2019-ncov/downloads/php/open-america/community-mitigation-quicklinks.pdf.

†† https://www.cdc.gov/coronavirus/2019-ncov/php/contact-tracing/contact-tracing-plan/contact-tracing.html.
